# Quality assessment of histopathological stainings on prolonged formalin fixed thrombus tissues retrieved by mechanical thrombectomy

**DOI:** 10.3389/fneur.2023.1223947

**Published:** 2023-12-13

**Authors:** Cem Bilgin, Daying Dai, Collin Johnson, Oana M. Mereuta, David F. Kallmes, Waleed Brinjikji, Ramanathan Kadirvel

**Affiliations:** ^1^Department of Radiology, Mayo Clinic, Rochester, MN, United States; ^2^Department of Neurologic Surgery, Mayo Clinic, Rochester, MN, United States

**Keywords:** ischemic stroke, histopathology, mechanical thrombectomy, thrombosis, formalin

## Abstract

**Background:**

Formalin-fixed retrieved clots from mechanical thrombectomy (MT) are now routinely studied using both conventional histopathologic techniques and immunohistochemistry (IHC). However, the effects of prolonged formalin fixation on the histological results of clot analysis remain unknown. The objective of this study was to investigate the effects of prolonged formalin fixation on quality of histopathologic stainings of thrombus tissues retrieved by MT.

**Methods:**

As part of the multicenter EXCELLENT registry, a total of 80 clots extracted by MT from acute ischemic stroke patients were randomly selected from the tissue database and assigned into four groups according to 10% neutral buffered formalin (NBF) fixation duration (1–30, 30–60, 60–90, and 90+ days, up to 2 years). Samples underwent processing and sectioning. Two serial sections for each case were stained with hematoxylin and eosin (H&E), Martius Scarlet Blue (MSB), and IHC for CD42b (platelet marker). An expert pathologist, who was blinded to tissue fixation duration and patient clinical data, assessed the quality of each stain including stainability, sensitivity, specificity, and consistency of stainings.

**Results:**

No significant issues were encountered during tissue processing and sectioning. On H&E stain, 97.5% (78/80) of slides showed good-quality staining, demonstrating clear histological properties of the thrombus tissue as red blood cells (RBC) stained in red, fibrin/platelet stained in pink, and nuclei stained in blue with intranuclear detail. The same histological features were also successfully demonstrated on MSB for all 80 samples. One of the 80 samples (1.2%) showed that RBC lost stainability on H&E due to tissue autolysis. Clear positive signal of platelet staining was expressed in 98.8% of the samples (79/80) with minimal background staining on IHC. There was no significant difference in staining quality across different formalin fixation groups.

**Conclusion:**

A good quality of histopathological staining is achievable for the thrombus tissue fixed in 10% neutral buffered formalin for up to 2 years. The findings are limited to the thrombus tissue retrieved by MT and specific fixation and staining protocols used in the study. To apply these results to other tissue or experimental setups, further studies and validations would be necessary.

**Clinical trial registration:**

This study was conducted as part of the EXCELLENT study: www.clinicaltrials.gov, unique identifier: NCT03685578.

## Introduction

The introduction of second-generation thrombectomy devices and the increased utilization of endovascular treatment for the emergent large vessel occlusions (ELVO) have allowed the histopathological analysis of the retrieved thrombus tissues. As a result, thrombus composition and its implications for thrombectomy procedures have gained an increasing amount of interest in recent years (1–4).

In the histological analysis of thrombus tissues, it is imperative to maintain the thrombus integrity close to its natural state. Fixative is used in histopathology to maintain clear and consistent morphological features, which is achieved by preventing destruction of the micro-architecture of tissue by stopping the activity of catabolic enzymes and autolysis. Neutral buffered formalin (NBF) of 10% (pH 7.2–7.4) is the most common fixative used in routine diagnostic pathology and is the fixative of choice in clot analysis (5, 6). NBF solution of 10% consists of sodium phosphate and 3.7% formaldehyde. Phosphate salts balance pH values, and formaldehyde is the essential fixative component of NBF. Formaldehyde forms inter- and intra-molecular covalent bonds and creates cross-links that primarily affect proteins and DNA (7, 8). Thus, it terminates biochemical reactions and prevents the enzymatic breakdown of clot components (7).

Formalin’s cross-linking is a continuous and progressive reaction. Therefore, prompt, appropriate, and adequate fixation of the tissue is essential to preserve cells and tissue components and to achieve optimal staining as well. Inadequate, poor fixation will result in color reduction, loss, or modification of hematoxylin and eosin (H&E) and histochemical stains. On the contrary, prolonged fixation will also cause the modification of stains due to changes of fixatives such as pH value. In terms of immunohistochemistry (IHC), poor fixation will cause the loss of antigenicity associated with tissue degradation. Nevertheless, excessive cross-linking associated with over-fixation will mask the antigenic epitopes which may result in irreversible loss of antigens. Hence, both conditions will negatively impact the immunohistochemical evaluation of the structure and function of cells and tissues (7–10).

The ideal sample fixation time is 24 h for histological clot analysis (6). However, clot analysis may not be feasible at the site of thrombectomy, and sample transportation to a histology core facility may be required. These clots are transferred in 10% NBF solution following standard operating procedures and protocols implemented by the histology core lab. Given the potential delays in sample transfer due to the ongoing COVID-19 pandemic, prolonged formalin fixation is inevitable in such cases. Despite its widespread use in histological clot analysis, an upper time limit for formalin fixation has not been determined yet. In this study, we sought to investigate the effects of prolonged formalin fixation on the histological stainings and IHC for histopathological evaluation.

## Methods

### Clot collection

This study was part of a prospective, multicenter, and international Embotrap Extraction and Clot Evaluation and Lesion Evaluation for Neuro Thrombectomy (EXCELLENT) registry (11). The study protocol was approved by the institutional review board at each institution (Supplementary Table S1), and written informed consent was obtained from enrolled patients (11). Patients were included in the study if they were ≥ 18 years, had undergone thrombectomy for ELVO, and had embolic material available for analysis. Patients with positive pregnancy tests were excluded.

### Histological clot analysis

A total of 80 clots were included in this study. Following MT retrieval, thrombus tissues were immediately fixed in 10% NBF and subsequently shipped to a histology core facility for processing. On arrival, clots were randomly assigned to four groups according to planned formalin fixation duration: group 1, 1–30 days (*N* = 20); group 2, 30–60 days (*N* = 20); group 3, 60–90 days (*N* = 20); and group 4, 90+ days, up to 2 years (*N* = 20). Thrombus tissues were further processed using a previously published, routine lab tissue processing protocol (12). Samples were embedded in paraffin blocks and cut into 3 μm sections. Serial sections were stained with H&E and Martius Scarlet Blue (MSB) following a lab routine staining protocol as described previously (12, 13). IHC was also performed for platelets marker (CD42b) on an autostainer (Leica Biosystems Ltd.; Nussloch GmbH, Germany; model Bond Max). Tris-EDTA was used for antigen retrieval. The sections were incubated with the primary antibody anti-CD42b (ab27669, 1:200 dilution) for 20 min. Additionally, negative controls were included and incubated with non-immune serum instead of primary antibody. The RedMap kit (Bond Polymer Refine Red Detection System, Leica Biosystems Ltd.) was used for visualization.

Tissue processing, sectioning, and all stains were performed by the same trained, experienced lab personnel.

### Quality assessment

An expert pathologist with 20 years of experience, blinded to clinical data and fixation duration, reviewed all stained slides and assessed the quality of stains included in the current study. The quality assessment was performed by evaluating the following standards for each stain.

#### H&E stain

Stainability is the most essential standard to reach in routine diagnosis pathology. On H&E, the cytoplasm, connective tissue fibers, and matrices are to be stained in pink or pink to red, and the cell nuclei are to be stained in blue or blue-black with intranuclear detail. The slides stained in different batches and the serial sections mounted on the same slide are to have the similar/same staining features mentioned above. The stained slides were considered to have a good quality if they met all these criteria.

#### MSB stain

Similar to H&E, stainability is the most essential goal to reach. On MSB stain, young fibrin and muscle are to be stained in red; red blood cells (RBCs) are to be stained in bright yellow or orange and easily discernable from associated tissue components; collagen and matured fibrin are stained in blue. Nuclei are to be stained in blue or blue-black with clear intranuclear detail. Tissues stained in different batches and serial sections on the same slide are to present the similar/same properties. The stained slides were considered as having a good quality if they met all the characteristics mentioned above.

#### Immunohistochemistry

Stainability, sensitivity, and specificity are the most important objectives of an IHC technique. (1) *Stainability* differences in the type and duration of fixative, variation in tissue processing, section thickness, reagents, and autostainer all are important factors to bring up the varying staining results from different laboratories. Development and validation of a staining protocol using laboratory’s own materials with a tissue containing a known targeted antigen is the key step to achieve a good quality IHC stain. A good IHC protocol is to achieve reproducible and consistent demonstration of antigen with maximum intensity and minimum background staining. Therefore, tissue slides with known antigen serving as a positive control in every staining run are required to evaluate the stain results. (2) *Sensitivity* refers to the relative amount of antigen that can be detected by an IHC technique. Detection of a small amount of antigen suggests that the protocol/technique has high sensitivity. (3) *Specificity* in IHC refers to an antibody that selectively binds to a single epitope on an antigen rather than any other protein. For an IHC staining to be specific, no staining should occur in the absence of the primary antibody. The tissue components that do not possess the target protein within the test sections should not present any staining. Therefore, a negative control involving the omission of the primary antibody from the staining procedure is essential to interpret the staining results. In summary, a good quality IHC staining should demonstrate the tissue of known positivity to be stained; the negative controls are to be negative; the positive signal is maximally projected with minimal background staining; minimal or absent variations occur between different batches, different staining runs, and serial sections as well.

### Statistical analysis

All statistical analyses were performed using IBM SPSS Statistics version 28. Categorical variables were compared using the Fisher–Freeman–Halton Exact test. A level of statistical significance for all analyses was set at a *p* < 0.05. Descriptive statistics were given for continuous variables. The first author had full access to all the data in the study and took responsibility for its integrity and the data analysis.

## Results

### Group 1 (1–30 days)

**Figure 1:**
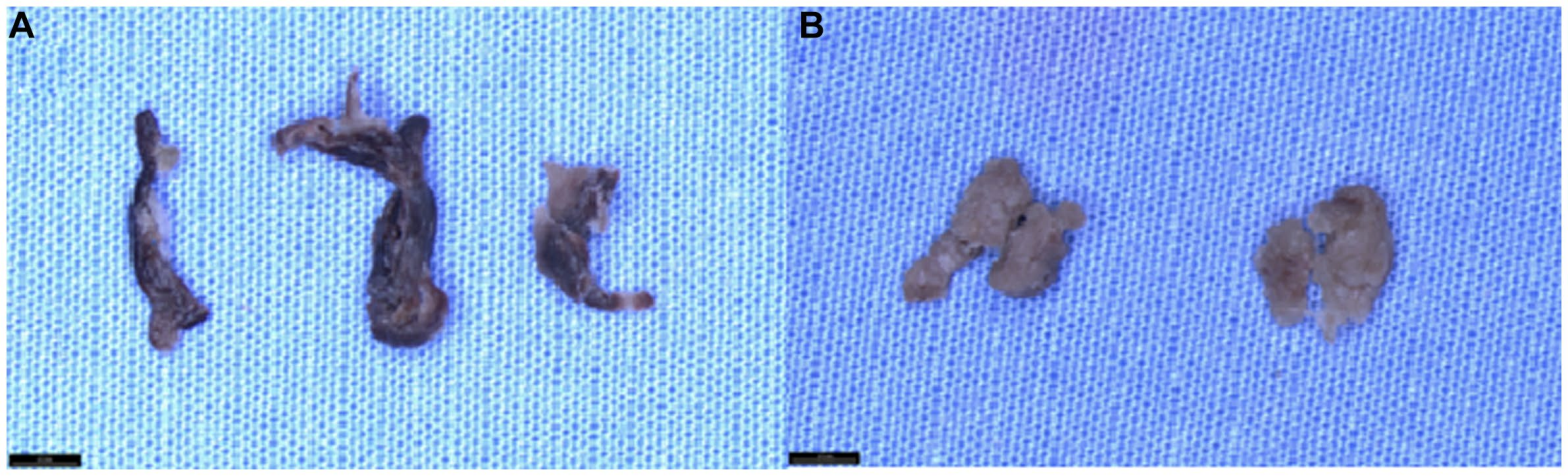
Gross appearance of clots at different time points following prolonged fixation. **(A)** The gross appearance of mixed clot tissue fixed in 10% neutral buffered formalin (NBF) at 1 month, 1 year, and 2 years (from left to right). Upon visual inspection, we observe that clot tissue’s color tone and texture do not undergo any significant changes during prolonged storage in 10%NBF. **(B)** The gross appearance of “white” clot tissue fixed in 10% neutral buffered formalin (NBF) at 1 month and 2 years (from left to right) demonstrates that the color tone and tissue texture of the clot tissue remain unchanged during prolonged storage in 10% NBF.

All 20 samples included in this group demonstrated good staining results on H&E and MSB (Figure 1). Each component was stained accordingly. RBCs were stained in red on H&E and in bright yellow/orange on MSB; fibrin/platelets were stained pink on H&E, and red (fibrin) or pink (platelets) on MSB; white blood cells and other nucleated cells were stained as blue or blue-black on both H&E and MSB with intranuclear detail. Platelets were maximally expressed with a clear staining signal in all 20 samples immunostained using the CD42b marker, without background staining (Figure 1).

The staining results of H&E, MSB, and IHC between different batches and different runs did not change. The results shown here suggest that thrombus tissues fixed in 10% NBF for 1 month did not cause difficulty for desired histologic stains.

### Group 2 (30–60 days)

**Figure 2:**
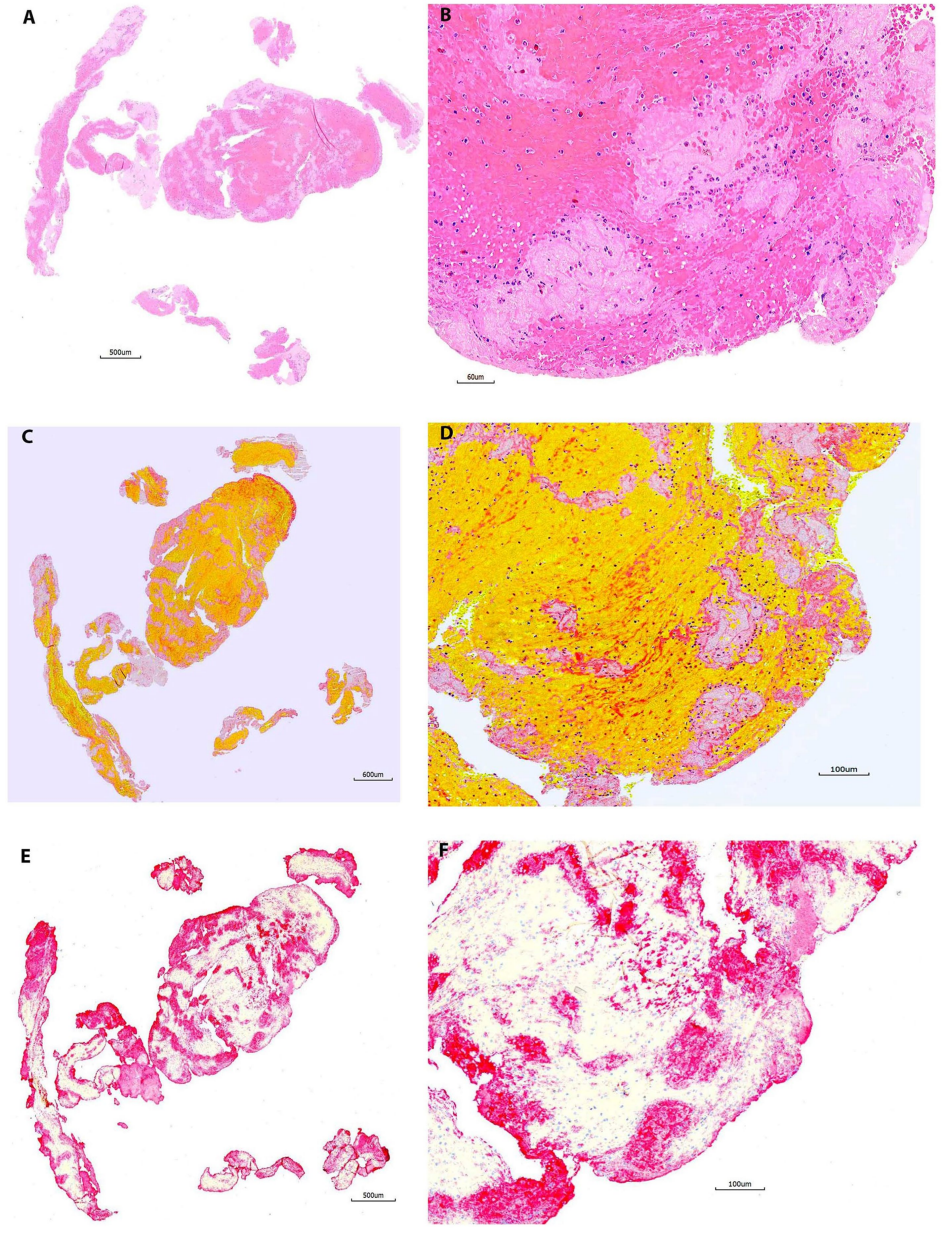
Representative microphotographs of group 1 (1–30 days). **(A,B)** Hematoxylin and eosin (H&E). **(C,D)** Martius Scarlet Blue (MSB). **(E,F)** Immunohistochemistry (IHC, antibody for CD42b, AP). **(A,C)**, and **(E)** are taken at lower magnification showing the overall staining results on entire section. **(B**,**D)**, and **(F)** are taken at higher magnification corresponding to **(A,C)**, and **(E)**, showing the tissue components on different stains. On H&E **(B)**, the red blood cells (RBCs) area is stained as red, the pink or light pink are fibrin/platelets. The nuclear cells are stained with blue. On MSB **(D)**, the RBCs are stained in yellow/orange, the fibrin is stained in red; the light pink area is platelets. Each component is easily recognized on both H&E and MSB. Platelets are clearly, maximally expressed with no background staining on IHC **(E,F)**, which corresponds well to that on H&E and MSB.

On H&E stain, 18 out of 20 samples (90%) showed good staining quality (Figure 2). The red color of fibrin/platelet stained with eosin appeared to be intense in one sample (5%), resulting in poor staining contrast between fibrin and RBCs. This section appeared to be thicker compared with sections on other slides included in the study. One out of twenty slides (5%) demonstrated that RBCs were stained in yellow instead of red. RBCs in this section appeared to melt down to become an aggregate without clear cells/tissue outline. As for MSB stain results in this group, all slides (100%) presented good staining quality similar to group 1 (Figure 2). One out of twenty slides (5%) showed an obvious difference in tissue color and staining density between the two serial sections stained with MSB. The two sections were placed on the slide far away from each other. The bottom section stained normally, while the top section showed different tissue color characteristics. The possible explanation could be that the level of reagents in the staining jar was too low to reach the top section, and consequently, the section was not properly stained by one or more dyes. Platelets were clearly projected on CD42b IHC in 19 out of 20 (95%) slides, with minimal background staining (Figure 2). The positive staining signal on one section was slightly weaker compared to the other section on the same slide in 2 out of 20 slides (10%). There are two possible reasons: (1) The section was thicker and contained tissue folds, an artifact that frequently occurs during sectioning; (2) the cover tile used was previously damaged (deformed, scratched, or cracked), and therefore, the antibody/solution was unequally dispensed on the slide during the staining.

### Group 3 (60–90 days)

**Figure 3:**
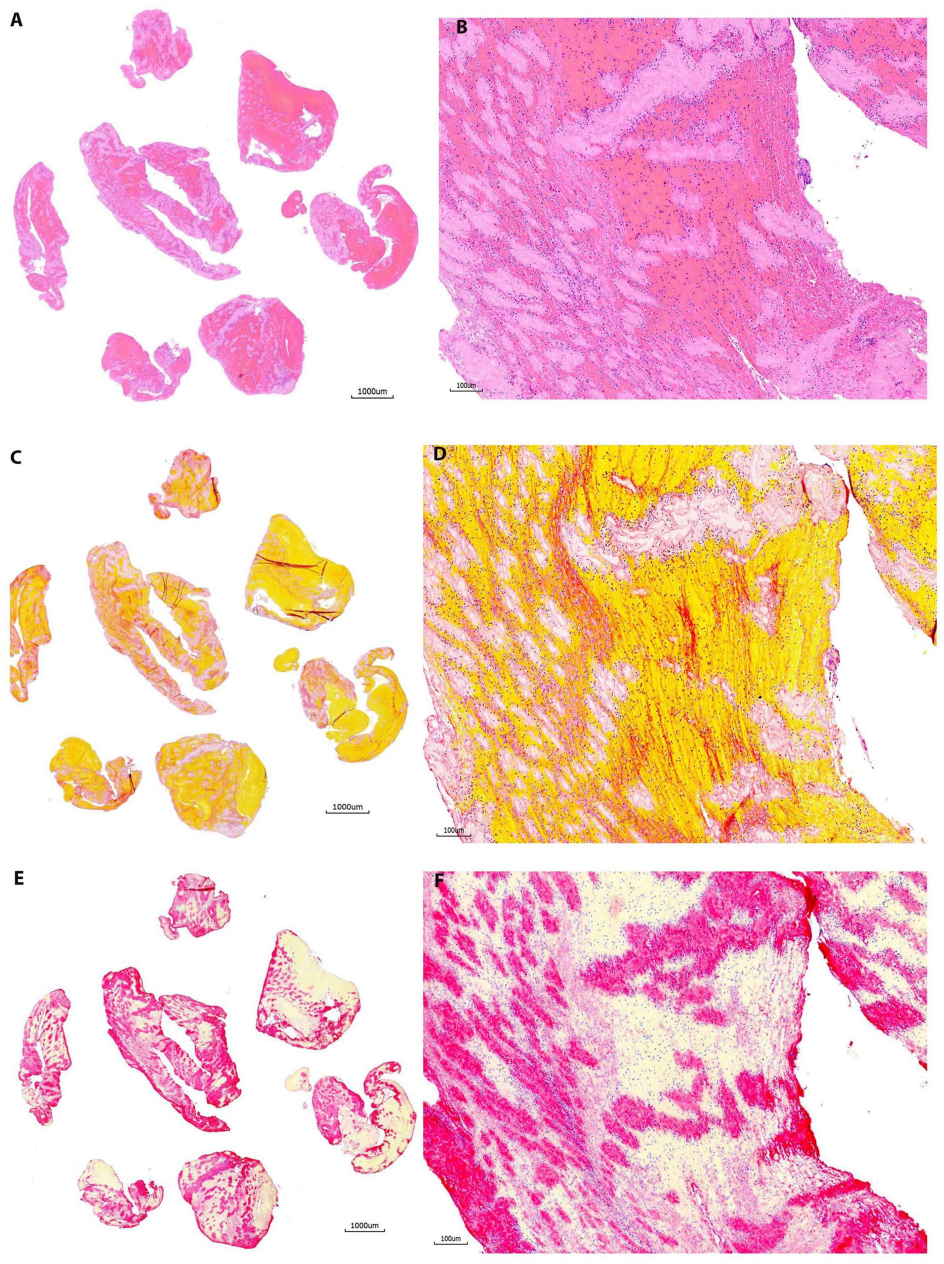
Representative microphotographs of group 2 (30–60 days). **(A,B)** Hematoxylin and eosin (H&E). **(C,D)** Martius Scarlet Blue (MSB). **(E,F)** Immunohistochemistry (IHC, antibody for CD42b, AP). **(A,C)**, and **(E)** are taken at lower magnification showing the overall staining results on entire section. **(B,D)**, and **(F)** are taken at higher magnification corresponding to **(A,C)**, and **(E)**, showing the tissue components on different stains. Each component is stained properly as mentioned in Figure 2.

All slides stained with H&E (20/20 [100%]) and MSB (20/20 [100%]) in this group demonstrated good staining results similar to groups 1 and 2 (Figure 3). All IHC-stained slides (20/20 [100%]) showed good staining results demonstrating maximum positive signal, with minimal background staining (Figure 3). The positive staining signal on one section was more intense compared to the other section on the same slide in one sample (1/20, 5%).

### Group 4 (>90 days)

**Figure 4:**
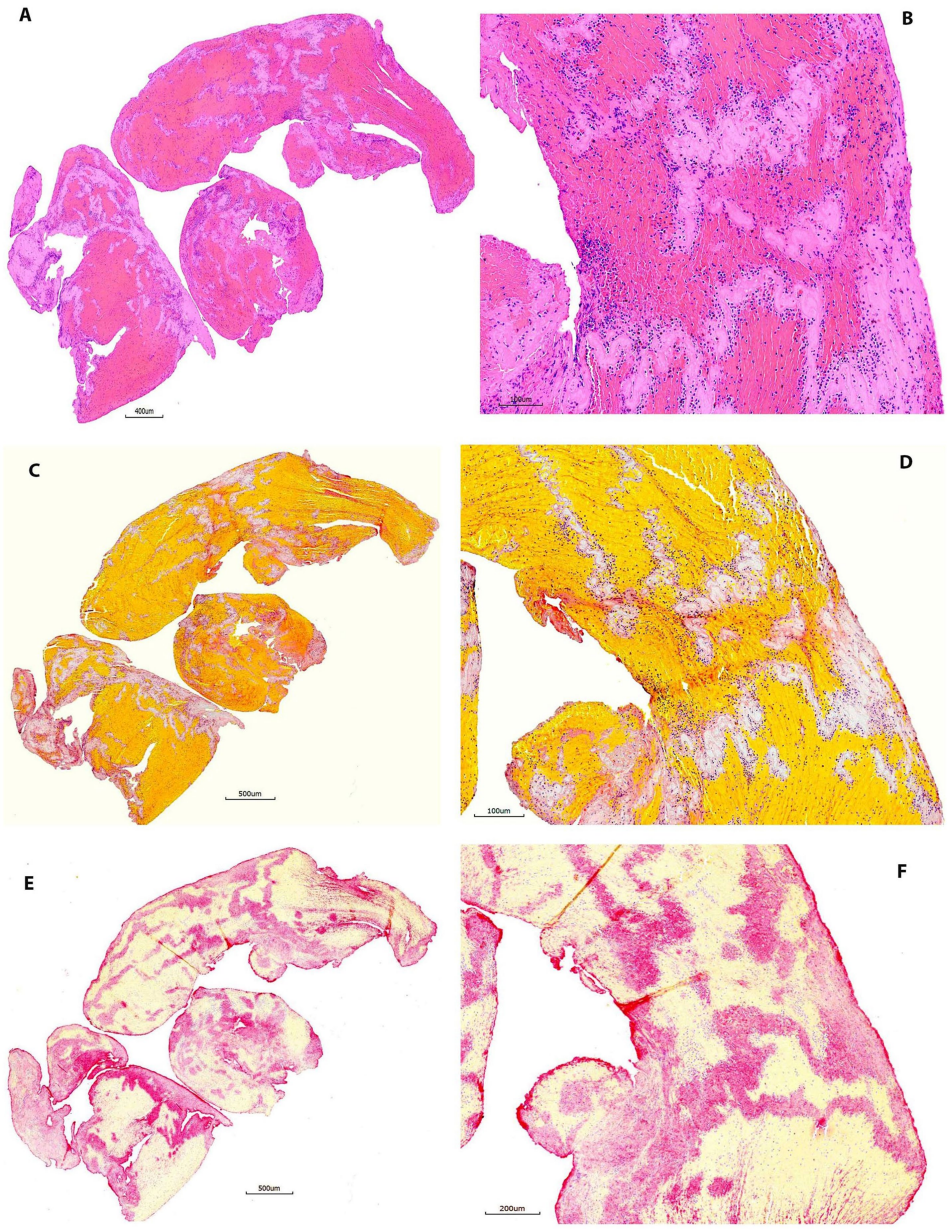
Representative microphotographs of group 3 (60–90 days). **(A,B)** Hematoxylin and eosin (H&E). **(C,D)** Martius Scarlet Blue (MSB). **(E,F)** Immunohistochemistry (IHC, antibody for CD42b, AP). **(A,C)**, and **(E)** are taken at lower magnification showing the overall staining results on entire section. **(B,D)**, and **(F)** are taken at higher magnification corresponding to **(A,C)**, and **(E)**, showing the tissue components on different stains. Each component is stained properly as mentioned in Figure 2.

All slides in this group showed good staining results on H&E, MSB, and IHC (20/20 [100%] for each stain) (Figure 4) similar to the previous groups. No significant abnormality was observed in these stainings. The results presented here suggested that thrombus tissues fixed in 10% NBF for more than 3 months did not cause difficulty for desired histologic stains.

### Comparison between the groups

The results were summarized in Tables 1, 2. Overall, 2 out of 80 samples (2.5%) showed either lost stainability of tissue components or reduced contrast between components on H&E. However, these differences in H&E stain quality were not statistically significant among groups (*p* = 0.24).

**Table 1:** Quality assessment of H&E and MSB staining formalin fixation.

	Formalin fixation duration					
	0–30 days	30–60 days	60–90 days	90+ days	Overall	*p*-values across groups
Outcome	*N*	*N*	*N*	*N*	*N* (%)	
**H&E stainability**						
Good	20	18	20	10	78 (97.5)	0.24
Poor	0	2	0	0	2 (2.5%)	
**H&E consistency**						
Good	20	20	20	20	80 (100)	N/A^*^
Poor	0	0	0	0	0	
**MSB stainability**						
Good	20	20	20	20	80 (100)	N/A^*^
Poor	0	0	0	0	0	
**MSB consistency**						
Good	20	19	20	20	79 (98.8)	
Poor	0	1	0	0	1 (1.2)	>0.99

Consistency evaluated in the table refers to the similarity of staining between serial sections on same slide. Consistency between different batches and different runs is also evaluated and remained well through the entire study. ^*^A *p*-value could not be calculated because H&E consistency was a constant. H&E, hematoxylin and eosin; MSB, Martius Scarlet Blue.

**Table 2:** Quality assessment of IHC with CD42b.

	Formalin fixation duration				
	0–30 days	30–60 days	60–90 days	90+ days	Overall
Outcome	*N*	*N*	*N*	*N*	*N* (%)
**Stainability**					
Good	20	19	20	20	79 (98.8)
Poor	0	1	0	0	1 (1.2)
**Sensitivity**					
Good	20	20	20	20	80 (100)
Poor	0	0	0	0	0
**Specificity**					
Good	20	20	20	20	80 (100)
Poor	0	0	0	0	0
**Consistency**					
Good	20	18	19	20	77 (96.3)
Poor	0	2	1	0	3 (3.7)

Consistency evaluated in the table refers to the similarity of staining between serial sections on same slide. Consistency between different batches and different runs is also evaluated and remained well through the entire study. IHC, immunohistochemistry.

Good quality of MSB stain was achieved in all 80 samples. There was no significant difference in stainability and staining consistency between the groups.

**Figure 5:**
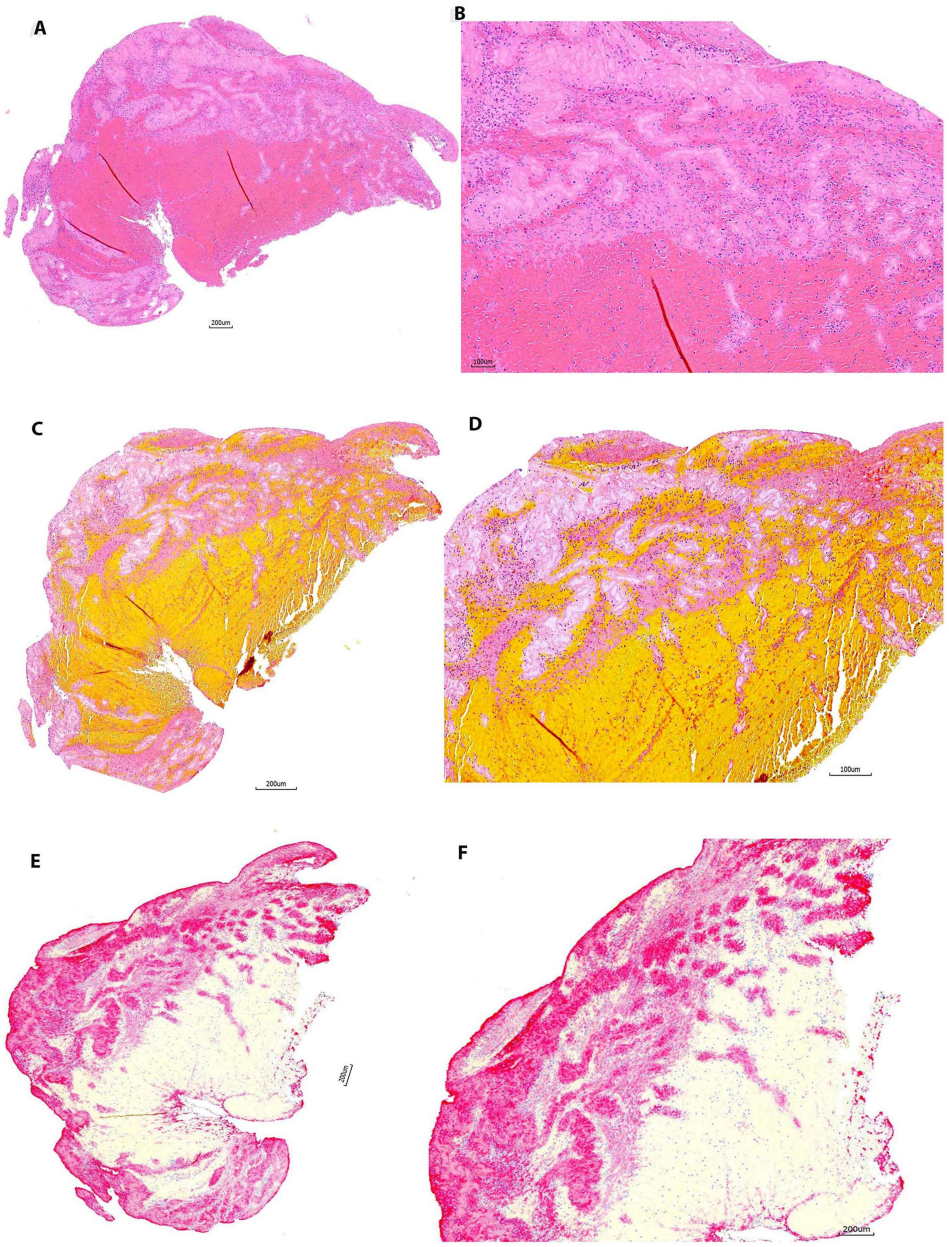
Representative microphotographs of group 4 (>90 days). **(A,B)** Hematoxylin and eosin (H&E). **(C,D)** Martius Scarlet Blue (MSB). **(E,F)** Immunohistochemistry (IHC, antibody for CD42b, AP). **(A,C)**, and **(E)** are taken at lower magnification showing the overall staining results on entire section. **(B,D)**, and **(F)** Are taken at higher magnification corresponding to **(A,C)**, and **(E)**, showing the tissue components on different stains. Each component is stained properly as mentioned in Figure 2. Good quality of stains is achieved after thrombus are fixed in 10% NBF more than 3 months.

The sensitivity, and specificity of CD42 IHC were satisfying in all samples (100%). The CD42 IHC stainability was poor only in one sample (98.8%, 1/80). In the study, 3 out of 80 samples (3.8%) had inconsistent staining results showing that one section was slightly weaker in intensity than the other section on the same slide. However, these differences in CD42b staining consistency were not statistically significant across groups (*p* = 0.61). Figure 1 compares the gross appearance of clots at 1 month and 2 years for different clot types. Figures 2–5 exhibit the representative microphotographs of each group.

## Discussion

In the current study, we showed that the quality of conventional histopathologic and histochemical stainings as well as IHC staining is well maintained for thrombus tissues fixed in 10% NBF for more than 3 months. These findings are important as they demonstrate and confirm that a good quality of histopathologic staining is achievable in tissue samples stored in 10% NBF over time. Untoward, unexpected delays in transferring biological tissue samples across institutions and countries, especially in the setting of the COVID-19 pandemic, will thus not degrade the potential data obtained from the analysis of clot samples.

The consequences of prolonged fixation on thrombus tissues retrieved from acute ischemic stroke patients have not been previously investigated. One study has examined how the prolonged 4% paraformaldehyde fixation affected immunofluorescence with clot analogs, showing autofluorescence became more evident with extended storage in paraformaldehyde (14). In addition, the authors used MSB stain to quantify the major clot components after fixation in paraformaldehyde for up to 2 months (14). However, the study did not include any immunohistochemistry (IHC) stain. Our study demonstrated that the quality of MSB staining remained good in all patients’ thrombus tissues fixed in 10% NBF for more than 3 months, up to 2 years. Additionally, we evaluated the quality of H&E staining in all tissues after prolonged fixation. Similar to MSB staining, good quality H&E stain was obtained in the majority of the samples (97.5%). In one sample (1.25%), RBCs lost their stainability with H&E. Morphologic examination showed RBCs in this sample were undergoing autolysis. The possible reason would be delayed fixation which can be avoided in future practice by promptly transferring the thrombus tissue into fixative once it is removed from the patient.

Prolonged fixation in formalin reduces or irreversibly damages the immunoreactivity of antigens resulting in poor or failed IHC staining. This aspect has been widely studied and reported over decades. The problems associated with formalin fixation in IHC have been dramatically reduced by introducing an antigen retrieval step in the staining procedure. The performance of IHC after prolonged fixation depends on two main factors, namely, the antigen retrieval technique and targeted antigen used (8, 10, 15). Antigen retrieval techniques are used to reverse the cross-linking of formalin and to improve the quality of immunostaining. The early phase of cross-linking is usually completed between 24 and 48 h and is mostly reversible at this stage (8). However, the number of cross-links increases with time, and this can make formalin’s effects irreversible despite the use of antigen retrieval techniques (7, 8). Studies suggest that some antigens are more sensitive to fixation time than others. For example, Paikrainen et al. showed that Amyloid-β immunostaining is feasible even after 14 years of fixation (10). Conversely, Arber showed that c-erb-B2 immunoreactivity could significantly change after 20 days of formalin fixation (9). Therefore, the effects of prolonged fixation should be investigated separately for each antigenic target; thus, protocol optimization is essential to achieve a good quality IHC stain and depends on the type of fixative, duration of fixation, as well as the antibody used.

CD42b is a platelet marker, widely used to identify the platelets in clot tissue (2). In contrast to MSB, CD42b immunostaining specifically detects platelets and can accurately distinguish platelets from other platelet-related proteins (1, 2, 6). In the present study, the application of antigen retrieval with EDTA-based solution at higher pH (pH 9) produced a clear, intense staining signal with minimal background in 79 samples (98.8%) fixed in 10% NBF over different time durations. The results suggested that long-term fixation in 10% NBF would not cause any difficulty in detecting the platelet component of thrombus tissues using IHC.

Overall, the issues we encountered are not related to the fixation duration but to other factors (such as microtomy, tissue mounted on the slides, and cover tiles used for IHC) which may require further assessment to ensure good quality of the stainings.

We found in a very small number of slides that one section had a weaker staining signal when compared with the other section on the same slide. This occurred in 1.2% of slides (1 out of 80) stained with MSB and 3.7% of the IHC slides (3 out of 80) in the current study. The possible causes could be as follows: (1) Sections were distributed too far away from the center of the slide, and therefore, the top section would be above the reagent level in the staining jar leading to the lack of staining during one or more steps of the H&E and MSB staining procedure. Moreover, the sections that were not placed at the center of the slide were outside the working field of the autostainer probe resulting in false-negative stain or an edge effect on IHC. This can be easily avoided by placing the sections at the center of the slide. (2) Difference in the thickness of serial sections on the same slide. The sections used in the current study were manually cut using a microtome, and, therefore, different speeds of sectioning and variations in the temperature of the paraffin block may result in different thicknesses of the serial sections. Cutting the desired number of sections at the same time, with the same speed, and keeping the block at a suitable temperature during sectioning by using an ice pad can efficiently avoid the differences in section thickness. (3) Some cover tiles used during IHC were damaged. The use of new cover tiles will eliminate this issue.

IHC staining sensitivity would be different on a different type of autostainer with a certain primary antibody; therefore, individual labs should optimize protocol using their own lab settings for each primary antibody to achieve the best staining result rather than “uniform.” Furthermore, lab personnel experienced in troubleshooting potential procedure problems are essential to provide effective and efficient quality control during the study.

Our study has limitations. (1) The sample size was small; only 10 samples for each stain were evaluated at the early time points. (2) Only one type of fixative (10% NBF) was evaluated in the presented study. We noticed in our daily practice that different types of fixative would largely impact the staining results on IHC even with the same primary antibody. Thus, we strongly suggest that the optimization of the staining protocol for individual labs should be achieved before starting any study. (3) The current study did not evaluate how the prolonged formalin fixation would affect the quality of immunofluorescence (IF), and, therefore, the findings related to the IHC stain presented in the current study might not apply to IF. It is important to note that current literature suggests that autofluorescence rates might increase with prolonged fixation (14). (4) Only one antibody marker (CD42b) was evaluated in the current study. It is well known that different antigens have different endurance limits of prolonged fixation; the findings regarding the CD42b IHC in the current study may not be applied to other antigens. (5) It is important to note that different cellular characteristics, such as blood type and clot composition, may the affect quality of histopathological outcomes, following prolonged formalin fixation (16).

## Conclusions

Good quality histopathological stainings can be achieved for the thrombus tissue fixed in 10% NBF for more than 3 months, up to 2 years. This suggests that the chosen fixation method is suitable for preserving the tissue and allows for successful staining even after an extended time period.

Effective and efficient quality control is the cornerstone for successfully accomplishing histopathological clot analysis.

## Data availability statement

The raw data supporting the conclusions of this article will be made available by the authors, upon reasonable request.

## Ethics statement

The studies involving humans were approved by the institutional review board at each institution (Supplementary Table S1). The studies were conducted in accordance with the local legislation and institutional requirements. The participants provided their written informed consent to participate in this study.

## Author contributions

CB, DD, CJ, OM, DK, WB, and RK: conception and design, acquisition of data, analysis and interpretation of data, drafting the article, critically revising the article, and administrative, technical, and material support. WB, DK, and RK: study supervision. All authors contributed to the article and approved the submitted version.
