# Supplementary material for: Quality assessment of histopathological stainings on prolonged formalin fixed thrombus tissues retrieved by mechanical thrombectomy
Source: Front Neurol. 2023 Dec 13;14:1223947. doi: 10.3389/fneur.2023.1223947 (PMC10751908; doi:10.3389/fneur.2023.1223947)
Supplement: Supplementary file 1 [file Table_1.DOCX]

**Supplemental Table 1. IRB Names**

| **Institution** **Name** **&** **Address** | **IRB/EC** **Name** **&** **Address** | **Clot** **Collection** |
| --- | --- | --- |
| Baptist Medical Center Lyerly Neurosurgery 800 Prudential Drive,  Tower B, 11th Floor Jacksonville, FL 32207 | The Institutional Review Board (IRB) of Baptist Health  820 Prudential Drive, Suite 413  Jacksonville, FL 32207 | Yes |
| Mount Sinai Hospital 1450 Madison Avenue New York, NY 10029 | Program for the Protection of Human Subjects  One Gustave L Levy Place Box 1081  New York, NY 10029 | Yes |
| University of Buffalo Medical Center  100 High Street, Suite 4B  Buffalo, NY 14203 | Western Institutional Review Board  1019 39th Avenue SE, Suite 120  Puyallup, WA 98374-2115 | Yes |
| Jackson Memorial Hospital  1611 NW 12^th^ Ave Miami, FL 33126 | University of Miami IRB 1400 NW 10^th^ Ave, 12^th^ Fl (M809)  Miami, FL 33136 | No |
| Norton Neuroscience Institute  4950 Norton Healthcare Blvd Suite 205, Louisville, KY  40241 | Western Institutional Review Board  1019 39th Avenue SE, Suite 120  Puyallup, WA 98374-2115 | No |
| University of Massachusetts Medical Center  55 N Lake Avenue Worcester, MA 01655 | University of Massachusetts, IRB 362 Plantation Street, Ambulatory Care Center, 7th Floor  Worcester, MA 01655 | No |
| Oregon Health and Science University 3181 SW Sam Jackson Park Road  Portland, OR 97239 | OHSU IRB  3181 SW Sam Jackson Park Road  Portland, Oregon 97239 | Yes |
| Cleveland Clinic Main Campus  9500 Euclid Avenue  Cleveland, OH 44195 | Cleveland Clinic IRB 9500 Euclid Avenue  Cleveland, OH 44195 | Yes |
| Vanderbilt University Medical Center  1211 Medical Center Dr  Nashville, TN 37232 | Human Research Protections Program  3319 West End Ave, Suite 600  Nashville, TN 37203 | Yes |
| Mercy Health Neuroscience  2222 Cherry St M200, Toledo, OH 43608 | Mercy Health North LLC IRB 2200 Jefferson Ave 4^th^ FL Toledo, OH 43604 | Yes |
| Fort Sanders Regional Medical Center, LLC 602 South Gay Street Suite 201C Knoxville, TN 37902 | Covenant Health IRB 280 Fort Sanders West Blvd  Building 4, Suite 204  Knoxville, TN 37922 | Yes |
| Los Robles Hospital and Medical Center 215 W Janss Rd, Thousand Oaks, CA  91360 | Western Institutional Review Board  1019 39th Avenue SE, Suite 120  Puyallup, WA 98374-2115 | Yes |
| Semmes-Murphey 6325 Humphreys Blvd  Memphis, TN 38120 | University of Tennessee Health Science Center  910 Madison Avenue  Memphis, TN 38163 | No |
| Texas Stroke Institute Medical City Plano 3901 W 15th St, Plano, TX 75075 | Medical City Plano IRB 3901 W 15th St, Plano, TX 75075 | Yes |
| OhioHealth Research Institute  3545 Olentangy River Rd # 301 Columbus, OH 43214 | Western Institutional Review Board  1019 39th Avenue SE, Suite 120  Puyallup, WA 98374-2115 | Yes |
| Grady Memorial Hospital  80 Jesse Hill Jr Dr SE, Atlanta, GA 30303 | Western Institutional Review Board  1019 39th Avenue SE, Suite 120  Puyallup, WA 98374-2115 | Yes |
| Jefferson Hospital for Neuroscience  900 Walnut St, Philadelphia, PA 19107 | Western Institutional Review Board  1019 39th Avenue SE, Suite 120  Puyallup, WA 98374-2115 | Yes |
| University of Tennessee Medical Center  1924 Alcoa Hwy,  Knoxville, TN 37920 | Sterling IRB 6300 Powers Ferry Rd  Suite 600-351  Atlanta, GA 30339 | Yes |
| Wellstar Kennestone Hospital  677 Church St,  Marietta, GA 30060 | Sterling IRB 6300 Powers Ferry Rd  Suite 600-351  Atlanta, GA 30339 | Yes |
| Memorial Regional Hospital  3501 Johnson Street  Hollywood, FL 33021 | Western Institutional Review Board  1019 39th Avenue SE, Suite 120  Puyallup, WA 98374-2115 | Yes |
| Geisinger Medical Center  100 N Academy Ave, Danville, PA 17822 | Geisinger IRB  100 N Academy Ave, Danville, PA 17822 | No |
| Banner Desert Medical Center  1520 S. Dobson Rd Ste 203 | Sterling IRB 6300 Powers Ferry Rd  Suite 600-351  Atlanta, GA 30339 | Yes |
| Mesa, AZ 85202 |  |  |
| Barrow Neurological Institute  350 West Thomas Rd  Phoenix, AZ 85013 | Sterling IRB 6300 Powers Ferry Rd  Suite 600-351  Atlanta, GA 30339 | Yes |
| Washington University in St. Louis  660 S. Euclid Ave, Campus Box 8057, St. Louis, MO, 63118 | Washington University in St.  Louis Human Research Protection Office  660 S. Euclid Ave, Campus Box 8089, St. Louis, MO 63110 | Yes |
| Advent Health Orlando 601 E. Rollins Ave, Mailbox 99, Neuroscience  Research, Orlando, FL, 32803 | Advent Health Orlando IRB 800 N. Magnolia Ave, Suite 500  Orlando, FL 32803 | Yes |
| Vidant Medical Center 2325 Stantonsburg Rd,  Greenville, NC, 27834 | East Carolina University IRB 4N-64 Brody Medical Sciences Building  600 Moye Blvd  Greenville, NC 27834 | No |
| Memorial Hermann – Texas Medical Center 6411 Fannin St  Houston, TX 77030 | UTHealth University of Texas IRB 6410 Fannin St, Suite 1100  Houston, TX 77030 | Yes |
| The University of Alabama at Birmingham  1720 Second Avenue  South, FOT 1007,  Birmingham, AL, 35294 | Sterling IRB 6300 Powers Ferry Rd  Suite 600-351  Atlanta, GA 30339 | Yes |
| University of Mainz Langenbeckstraße 1,  55101 Mainz, Germany | Ethikkommission Der Landesarztekammer Rheinland- Pfalz  Deutschhausplatz 3  55116 Mainz, Germany | Yes |
| AZ Groeninge President Kennedylaan 4  8500 Kortrijk Belgium | Comissie Medische Ethiek AZ Groeninge  President Kennedylaan 4  8500 Kortrijk Belgium | No |
| Hopital Roger Salengro – CHU Lille Avenue du Professeur Emile Laine  59037 Lille, France | Comite de Protection des Personnes Sud-Est III/CPP Sud- Est II  Groupement Hospitalier Est – Batiment Pinel  59 Boulevard Pinel  69500 Bron FranceN/A | No |
| Universitaetsklinikum Hamburg Eppendorf W14 Martinistrasse 52  20246 Hamburg Germany | Ethik-Kommission der Arztekammer Hamburg Weidestrasse 122 b  22083 Hamburg Germany | Yes |
| Klinikum Dortmund gGmbh Beurhausstrasse 40  44137 Dortmund Germany | Ethik-Kommission der Arztekammer Westfalen-Lippe und der Westfalischen Wilhems Universitat Munster Gartenstraße 210-214  48147 Munster, Germany | Yes |
| Universitätsklinikum des Saarlandes,  Kirrberger Strasse Gebäude 90, Homburg  Saar, 66421, Germany | Ethik-Kommission bei der Ärztekammer des Saarlandes,  Faktoreistraße 4, 66111 Saarbrücken,  Germany | No |
| Hadassah Medical Center,  Kiryat Hadassah, POB 12000, Jerusalem,  91120, Israel | EC Hadassah Hebrew University Medical Center,  Einkerem, POB 12000,  Jerusalem, 91120, Israel | No |
| Charing Cross Hospital,  Fulham Palace Road, Hammersmith, London, W6 8RF, United Kingdom | NRES Committee London - South East,  Barlow House, 3rd Floor, 4 Minshull Street, Manchester, M1 3DZ, United Kingdom | Yes |

IRB=Institutional Review Board, EC=Ethics Committee
